# Mapping the premigration distribution of eastern Monarch butterflies using community science data

**DOI:** 10.1002/ece3.7912

**Published:** 2021-07-14

**Authors:** Iman Momeni‐Dehaghi, Joseph R. Bennett, Greg W. Mitchell, Trina Rytwinski, Lenore Fahrig

**Affiliations:** ^1^ Department of Biology Carleton University Ottawa ON Canada; ^2^ Wildlife Research Division National Wildlife Research Centre Environment and Climate Change Canada Ottawa ON Canada

**Keywords:** citizen science, community science, *Danaus plexippus*, Journey North, Monarch butterfly, premigration distribution, sampling effort correction

## Abstract

Knowing the distribution of migratory species at different stages of their life cycle is necessary for their effective conservation. For the Monarch butterfly (*Danaus plexippus*), although its overwintering distribution is well known, the available information on premigration distribution is limited to the studies estimating the natal origins of overwintering Monarchs in Mexico (i.e., postmigration data). However, the premigration distribution and the natal origins of overwintering Monarchs can be equivalent only if we assume that migrating Monarchs have the same mortality rate irrespective of their origins. To estimate Monarchs’ premigration distribution, we used data reported by community scientists before Monarchs start their fall migration, that is, before migration mortality, and controlled for sampling bias. Our premigration distribution map indicated that Minnesota, Texas, and Ontario are the states/provinces with the highest abundance of Monarch in North America. Although this higher estimated abundance can be related to the large sizes of these states/provinces, this information is still important because it identifies the management jurisdictions with the largest responsibility for the conservation of the premigration population of Monarchs. Our premigration distribution map will be useful in future studies estimating the rates, distribution, and causes of mortality in migrating Monarchs.

## Graphical abstract

Our paper provides the first quantitative estimate of the premigration distribution of Monarch butterflies across North America. Our results showed that Minnesota, Texas, and Ontario are the states/provinces with the highest abundance of Monarchs before they start their fall migration. This information is important because it identifies the management jurisdictions with the largest responsibility for the conservation of the premigration population of Monarchs. Our premigration distribution map will be useful in future studies estimating the rates, distribution, and causes of mortality in migrating Monarchs.

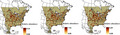

## 1 INTRODUCTION

Although migration has many advantages, it also has risks (Pimm et al., 1988; Rankin & Burchsted, 1992). In comparison with sedentary species, migratory species have greater annual population fluctuations (Pimm et al., 1988; Vickery et al., 2014; Wilcove & Wikelski, 2008), partly due to their more complex annual life histories. Migratory species must transition through more life history stages than sedentary or resident species, each of which is time‐constrained (Wingfield, 2008). Migratory species also depend on multiple habitat types throughout their annual cycle, varying in availability (Runge et al., 2014). The first step to understanding migratory species’ habitat requirements and factors affecting their vital rates is understanding their distribution in space and time.

The annual migration of the eastern population of the Monarch butterfly (*Danaus plexippus*) has long captured the public's imagination. Every year around mid‐August, millions of Monarch butterflies migrate to Mexico from their breeding regions in Canada and the United States, a distance of up to 3,500 km. They overwinter in oyamel fir (*Abies religiosa*) forests in the mountains west of Mexico City, and from the start of spring, they begin a northward migration to their breeding regions.

In recent years, the eastern Monarch's population size, as estimated on the wintering ground, has declined considerably (Semmens et al., 2016; Vidal & Rendón‐Salinas, 2014). This decline is so sharp that the United States Fish and Wildlife Service considered Monarch butterfly as a candidate for listing under the Endangered Species Act (www.fws.gov/savethemonarch/SSA.html), and the Committee on the Status of Endangered Wildlife in Canada (COSEWIC) has recommended uplisting the Monarch in Canada from a species of Special Concern to an Endangered species. Numerous threats may affect the Monarch butterfly population size, including wintering habitat loss (Vidal & Rendón‐Salinas, 2014), climate change (Oberhauser & Peterson, 2003), effects of pesticides and herbicides on nectaring plant and milkweed availability (Flockhart et al., 2015; Pleasants & Oberhauser, 2013; Thogmartin et al., 2017), disease (Satterfield et al., 2015), climate extremes (Agrawal & Inamine, 2018; Crewe et al., 2019), and roadkill (Kantola et al., 2019; Mckenna et al., 2001; Mora Alvarez et al., 2019).

Although monarch populations continue to decrease, and the main decline is thought to be driven by habitat loss on the breeding grounds (Flockhart et al., 2015), indirect evidence suggests migration‐related mortality does affect the wintering population size (Saunders et al., 2019). Moreover, to date, expert opinion (Flockhart et al., 2015; Oberhauser et al., 2017) suggests high rates of migration‐related mortality and substantial regional differences in mortality rates that vary across latitude and longitude. However, there is little empirical evidence to date of regional variation in migration‐related mortality (but see Taylor et al., 2020) or of the causes of this mortality. The unknown distribution of Monarchs in late summer, just before Monarchs start their fall migration, is one of the major limiting factors for studies related to migration‐related mortality. There are some studies of the natal origins of Monarchs using data collected after migration to Mexico (Flockhart et al., 2013, 2017; Hobson et al., 1999, 2019; Wassenaar & Hobson, 1998). These studies use methods such as stable‐hydrogen, carbon, and oxygen isotope measurements. However, the natal origins of overwintering Monarchs can be equivalent to the premigration distribution of Monarchs only if we assume that migrating Monarchs have the same mortality rate irrespective of their origins. Moreover, available maps of tagging efforts (Taylor et al., 2020) and species distribution models (Batalden et al., 2008; Castañeda et al., 2019; Flockhart et al., 2019; Lemoine, 2015) also do not necessarily accurately indicate Monarchs’ premigration distribution.

In this paper, our goal was to use data generated from community science (a.k.a. citizen science) to estimate the premigration distribution of Monarch butterflies across eastern North America. The resulting map can be used to estimate jurisdictional (state/province) responsibilities with respect to conservation of the breeding population, to estimate breeding population size in the United States and Canada, and to direct a wide range of future research addressing Monarchs mortality.

## 2 METHODS

### 2.1 Overview

To estimate the premigration distribution of the eastern migratory population of Monarchs, we used data from community science monitoring, adjusted for sampling effort. We used all adult Monarch sightings reported in the Journey North community science program (www.journeynorth.org) within the distribution of the eastern migratory population from 15th July to 15th August of 1996–2020. Due to data sparseness in some regions, we grouped the sightings into 899 coarse pixels. To correct for unequal sampling effort, we then adjusted the numbers based on the number of observers per pixel, resulting in a premigration distribution map of Monarchs in North America. Finally, we summarized the premigration distribution map based on different states/provinces.

### 2.2 Delineation of the eastern migratory population

**FIGURE 1:**
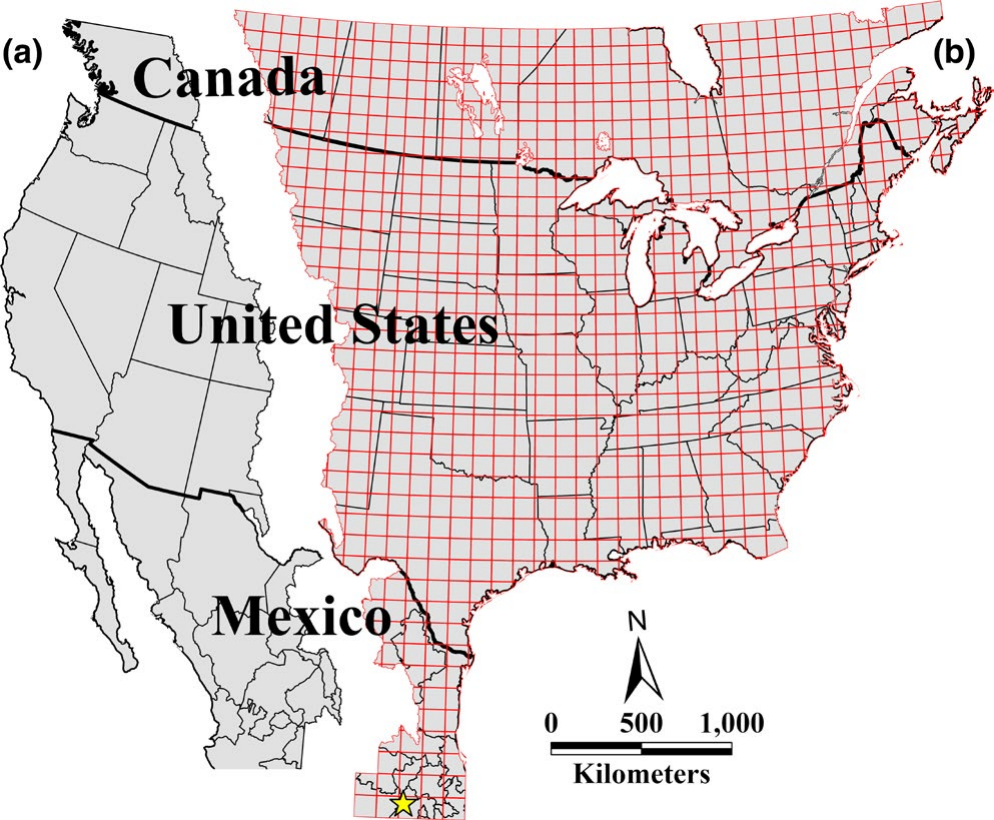
Delineation of the potential extent of the eastern migratory population of Monarchs. The Rocky Mountains separate the western (a) and eastern (b) populations. Monarchs in southern Florida generally do not migrate to the overwintering site in Mexico (yellow star) and are therefore not considered part of the population. We divided the study area into 899 100 × 100 km pixels

Our study area covered the potential extent of the eastern migratory population of Monarchs. The Rocky Mountains separate the eastern and western populations of Monarch butterflies (Brower, 1995). We used the digital layer of North America's watersheds (CEC, 2010) to draw the ridgeline of the Rocky Mountains as the western limit (Figure 1). We used the highest latitude that a Monarch has been observed as the northern limit and the Monarch Butterfly Biosphere Reserve (MBBR) in Mexico, where the population overwinters, as the southern limit. We excluded Southern Florida because many Monarchs in this region breed and overwinter locally and do not migrate to Mexico (Knight & Brower, 2009).

### 2.3 Sighting data

For sighting data, we needed a dataset that covers all of North America, has a large number of records for adult Monarchs, and, in addition to location, provides information about the number of Monarchs observed at each record. We found that the Journey North community science program (www.journeynorth.org) is the only dataset that meets all of these criteria. Although this program's primary goal is not recording the abundance of Monarchs, it provides all the information we needed for our research. The eButterfly (www.e‐butterfly.org) is another dataset that has data for both location and the number of Monarchs observed at each sighting. However, the spatial range of eButterfly dataset is limited to the United States and Canada, and we also required data for Mexico. Also, the number of sightings reported in eButterfly is considerably less than in Journey North (Figure S1). As these two datasets inherently are different in terms of the sampling effort and the spatial coverage, we avoided mixing them and limited our data to only the Journey North data. We used Journey North data recorded from 15th July to 15th August in all available years (1996–2020), to coincide with the period just before the start of migration toward the overwintering habitat in Mexico, in mid‐August (Gibo & McCurdy, 1993). In cases of multiple sightings for the same exact location and date, we randomly selected one sighting and excluded the others from further analysis. In total, 10,597 sightings remained for the next steps.

### 2.4 Correcting for bias

As the Journey North database is based on opportunistic sightings by citizens, it suffers from a highly uneven sampling effort relative to the underlying distribution of Monarchs, with more sightings in the regions with higher human population densities. In some parts of the study area, sampling effort is very sparse. To reduce the number of pixels with no sightings, we amalgamated the data into 899, 100 × 100 km pixels and clipped the resulting layer based on our study area (Figure 1b).

We assumed that the human population and the number of observers contributing to reported Monarchs in a pixel are two indices of sampling effort. Therefore, we calculated the average human population density (CIESIN, 2018) and the number of unique observers in each pixel. Using the “MASS” package (Venables & Ripley, 2002), for the cells with at least one reported sighting, we fitted three negative binomial models predicting the total number of sightings in each pixel, on (1) human population density, (2) number of unique observers, and (3) human population and number of observers. To find the link function that best fit our data, we used the “caret” package (Kuhn, 2020). Because the two predictors we used in the models are positively correlated (*r* = .75), we selected the single‐predictor model with the lowest Root Mean Square Error (RMSE) and the highest *R* ^2^ value. Note that sightings are individual records of Monarch presence; actual abundances observed vary among sightings. We then calculated the ratio of the observed number of sightings divided by the predicted number of sightings per cell, which indicates how many times the number of sightings per pixel is larger or smaller than the number of sightings predicted based on sampling effort. We considered the calculated ratio as a relative, bias‐corrected number of sightings per pixel.

To estimate the relative abundance of Monarchs for each pixel, we then multiplied the bias‐corrected number of sightings by the median number of Monarchs per sighting (ranging from 1–100) for that pixel. We note that this represents a relative, not actual, population size. To account for the uncertainty in our analysis, we calculated the confidence intervals for the predictions we made from the negative binomial model using the ciTools package (Haman & Avery, 2020) and repeated the above‐described process for estimating the relative abundance of Monarchs based on lower and upper bounds of the calculated confidence intervals. For the cells without any sightings, we considered three different scenarios, including: (1) In cells with no observations, the abundance is assumed to be zero; (2) in cells with no observations, the abundance is assumed to equal the minimum abundance in all the cells located in the corresponding breeding region as defined by Flockhart et al. (2017); and (3) in cells with no observations, the abundance is assumed to equal the median Monarch abundance in all the cells located in the corresponding breeding region.

We then converted the relative Monarch abundances into estimated probabilities by dividing each pixel's value by the sum of all the pixels’ values and calculated the percentage of Monarchs starting their migration from different provinces/states in our study area.

## 3 RESULTS

### 3.1 Relative abundances

**FIGURE 2:**
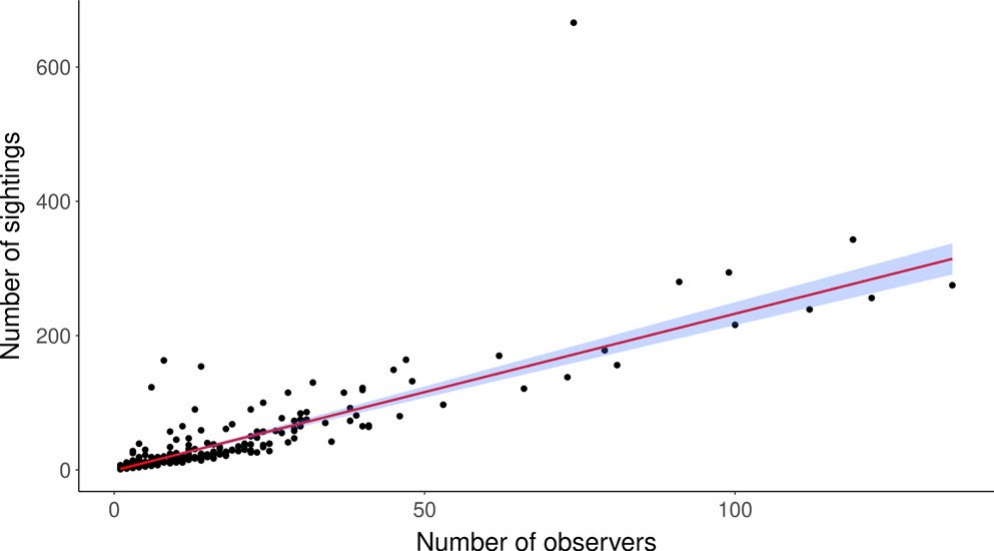
The positive relationship between the total number of sightings per pixel and the number of unique observers per pixel. Shown is the best‐fit line using a negative binomial model, with its 95% confidence interval. We used this model to determine how often the number of sightings per pixel is larger or smaller than the number of sightings predicted based on the number of observers. The resulting ratio was considered a bias‐corrected relative number of sightings per pixel

**FIGURE 3:**
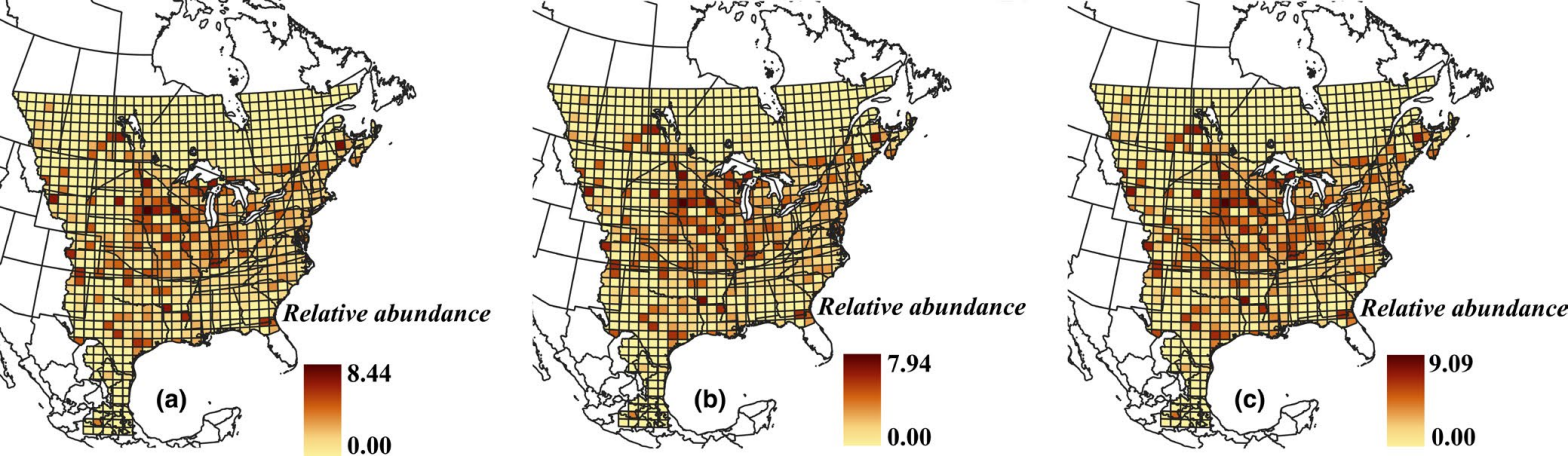
Premigration distribution of adult Monarch butterflies across North America based on the second scenario for addressing cells with no observation (i.e., abundance is assumed to equal the minimum abundance in all cells located in the corresponding breeding region as defined by Flockhart et al., 2017). (a) Relative abundances based on the mean number of Monarchs predicted per pixel, as a proportion of the total of all mean values across all pixels. (b) Relative abundances based on the minimum number of Monarchs predicted per pixel from the lower limits of the CI for each pixel, as a proportion of the total of all minimum values across all pixels. (c) Relative abundances based on the maximum number of Monarchs predicted per pixel from the upper limits of the CI for each pixel, as a proportion of the total of all maximum values across all pixels

Comparison of RMSE and *R* ^2^ values of different models showed that the number of unique observers per pixel was by far a better predictor than the human population per pixel and performed as well as the model combining both measures for estimating the number of sightings per pixel (Table S1). Therefore, we used the number of unique observers to correct for sampling effort; the relationship is shown in Figure 2. This comparison also indicated that the identity link is the best link function for our data. The premigration distribution map based on the second scenario for addressing cells with no observation (i.e., abundance is assumed to equal the minimum abundance in all cells located in the corresponding breeding region as defined by Flockhart et al. (2017)) and its confidence interval are shown in Figure 3a–c. The premigration distribution maps that resulted from the first and third scenarios for addressing cells with no observation are provided in Figure S2.

We found that Minnesota (13.1%–13.6%), Texas (5.6%–5.9%), and Ontario (5.0%–5.4%) are the states/provinces with the highest abundances of Monarchs from 15th July to 15th August (Tables 1 and S2).

**TABLE 1:** Five provinces/states with the highest relative abundance of adult Monarchs calculated by the second scenario. The complete list is provided in the Table S2

Province/State	Minimum premigration distribution (%)	Mean premigration distribution (%)	Maximum premigration distribution (%)
Minnesota	13.08	13.31	13.60
Texas	5.63	5.75	5.91
Ontario	5.01	5.23	5.37
Michigan	4.26	4.40	4.52
Wisconsin	3.90	4.07	4.21

## 4 DISCUSSION

Our analysis provides an estimate of the premigration distribution of Monarch butterflies that is not influenced by differential mortality during migration or on the wintering grounds. Before our study, all the available information on the premigration distribution of Monarchs in the breeding regions came from studies estimating the natal origins of overwintering Monarchs (Flockhart et al., 2013, 2017; Hobson et al., 1999, 2019; Wassenaar & Hobson, 1998). Such analyses make the key assumption that migration mortality is uniform throughout the Monarch's range and on the wintering grounds, irrespective of breeding location or migratory path. This assumption is unlikely to be the case, as the suit of factors contributing to Monarch migration mortality—for example, roadkill, extreme climate, predation, loss of nectar resources, and disease—will vary with the starting location and migration timing, which is also likely correlated with starting location.

Among three different scenarios we used to map the premigration distribution of Monarchs, we believe the second scenario—minimum abundance in each breeding region assigned to cells with no observations—provides more realistic estimates in comparison to the other two scenarios. The first scenario assumes that Monarch abundance is zero in the cells with no sightings. However, lack of sighting in a cell can be caused by either lack of Monarchs or lack of sampling effort. On the other hand, our third scenario—assigning cells with no observations a relative abundance equal to the median of Monarch relative abundance in the corresponding breeding region—likely overestimates the relative abundances in the cells with no sightings. More specifically, when we visually compared the premigration distribution map made by the third scenario to the natal origins maps of overwintering Monarchs (Flockhart et al., 2017; Hobson et al., 2019), and distribution maps resulted from SDMs (Flockhart et al., 2019; Lemoine, 2015), the overestimation in our map was noticeable, especially in the western regions.

Our premigration distribution map suggests that the proportion of Monarchs starting their migration from Minnesota, Texas, and Ontario is higher than for other states/provinces. This is likely mainly due to the large sizes of these states/provinces. Nevertheless, this information is important because it identifies the management jurisdictions with the largest responsibility for the conservation of the premigration population of Monarchs.

Although a premigration distribution map is critical for directing conservation actions, it is important to note that our methods and data have limitations. For example, there are many more reported sightings in the eastern regions of the study area than the western regions due to higher human (and observer) density in the east. Although we controlled for this spatial bias by including the number of unique observers in our models, the small number of sightings reported in the west still increases the uncertainty of our estimates of the relative abundance of Monarchs in western regions. To account for uncertainty in our analysis, we estimated the confidence interval of the negative binomial model in our predictions. However, there are other sources of uncertainty that we were not able to consider. For example, we could not consider the confidence interval around the calculated median number of Monarchs observed per pixel, because our low sample size in many pixels made it impossible to calculate parameters for statistical distributions for individual pixels. Also, community engagement may vary over time and space. In our map, instead of just using observations reported in recent years, which minimizes the risk of a shift in community engagement over time, we decided to keep the largest number of records by maximizing the period over which Monarchs were observed. As the distribution of Monarchs in North America varies considerably over years due to natural variation (Flockhart et al., 2019), it is not possible to create a reliable distribution map using data selected from a short period of time. As mentioned above, variation in community engagement through space and time is another source of uncertainty that we could not account for, because there are no data on each observer's effort. Similarly, we were not able to account for observer experience in our model because this variable was highly correlated with the number of observers per pixel (Appendix S1).

Our premigration distribution map will permit future studies to estimate some important parameters in Monarch butterfly population dynamics that were previously not estimable. For example, by comparing our estimates of premigration relative abundance to the estimates on postmigration data (Flockhart et al., 2017; Hobson et al., 2019), we can obtain estimates that might indicate the relative migration mortality among regions. Also, by knowing the premigration distribution of Monarchs, the population size of overwintering Monarchs and their natal origins, with some estimates of migration mortality, we should be able to estimate the actual population size of Monarchs in the breeding regions. The premigration distribution map might also be used to estimate the mortality rate of migratory Monarchs due to particular causes. For example, one could estimate mortality due to roadkill by simulating the migration of Monarchs toward Mexico based on the premigration distribution (i.e., the higher relative abundance in a pixel, the higher probability that a Monarch starts its migration from that pixel) and then calculating the probability of roadkill for Monarchs starting their migration from different regions. By comparing the adult premigration distribution to the distribution of Monarch's eggs and larvae, one could identify the regions that are mortality hotspots for Monarchs before they start their migration. Last, our premigration distribution map can be used to understand jurisdictional responsibility with respect to the management and conservation of Monarchs across the breeding range.

## CONFLICTS OF INTEREST

The authors confirm that there is no conflict of interest to declare.

## AUTHOR CONTRIBUTIONS

**Iman Momeni‐Dehaghi:** Conceptualization (equal); Formal analysis (lead); Investigation (equal); Methodology (equal); Software (lead); Visualization (lead); Writing‐original draft (lead). **Joseph R. Bennett:** Conceptualization (equal); Investigation (lead); Methodology (equal); Supervision (lead); Validation (equal); Writing‐review & editing (equal). **Greg W. Mitchell:** Conceptualization (equal); Investigation (equal); Methodology (equal); Supervision (equal); Validation (lead); Writing‐review & editing (equal). **Trina Rytwinski:** Conceptualization (equal); Investigation (equal); Supervision (equal); Validation (equal); Writing‐review & editing (equal). **Lenore Fahrig:** Conceptualization (lead); Investigation (equal); Methodology (equal); Supervision (lead); Validation (equal); Writing‐review & editing (equal).

## Supporting information

Appendix S1‐S3

## Data Availability

The sightings dataset is available on Journey North website: https://journeynorth.org/sightings/. The human population density raster is available on Columbia University Web repository: https://beta.sedac.ciesin.columbia.edu/data/set/gpw‐v4‐population‐density‐adjusted‐to‐2015‐unwpp‐country‐totals/data‐download.
