# Supplementary material for: Mapping the premigration distribution of eastern Monarch butterflies using community science data
Source: Ecol Evol. 2021 Jul 14;11(16):11275–81. doi: 10.1002/ece3.7912 (PMC8366871; doi:10.1002/ece3.7912)
Supplement: Supplementary file 1 — Appendix S1‐S3 [file ECE3-11-11275-s001.docx]

Supplementary information for Mapping the pre-migration distribution of eastern Monarch butterflies using community science data 

----------------------------------------------------------------------------------------------------
Appendix 1: Accounting for observer experience in model
----------------------------------------------------------------------------------------------------
To account for the observer experience in our model, we added a variable showing the total number of years that observers in each pixel contributed to the program. For this, we first calculated the number of years each observer contributed to reporting observed Monarchs in each pixel. Then, as a proxy of observer experience in each pixel, we summed the number of years that all the observers in each pixel contributed to reporting their observations. However, as a result of the high correlation between the calculated observer experience and the number of unique observers (r=0.987), we decided to exclude the observer experience variable and use the number of unique observers as the only predictor variable in our model. 


-------------------------------------------------------------------------------------------------------------
Appendix 2: Tables
----------------------------------------------------------------------------------------------------
Table S1 Model performance when the number of sightings per pixel was predicted based on human population (hp) and the number of unique observers (nouo) per pixel. RMSE: root-mean-square deviation.
Predictor	link	RMSE	R-squared
hp + nouo	identity	23.92	0.80
	log	13297.82	0.33
	sqrt	133.68	0.69
hp	identity	40.11	0.45
	log	2.67e+07	0.19
	sqrt	145.28	0.35
nouo	identity	27.21	0.77
	log	8307.82	0.29
	sqrt	133.52	0.66


Table S2 Relative abundance of adult Monarchs across provinces/states in the study area based on the second scenario.
Country	Province/State	Predicted relative abundance	CI
Canada	Alberta	2.290	2.222 - 2.342
	Manitoba	3.281	3.117 - 3.496
	New Brunswick	1.924	1.878 - 1.961
	Nova Scotia	0.779	0.777 - 0.782
	Ontario	5.226	5.029 - 5.375
	Prince Edward Island	0.549	0.517 - 0.590
	Quebec	1.431	1.426 - 1.435
	Saskatchewan	1.752	1.674 - 1.854
US	Alabama	0.499	0.491 - 0.506
	Arkansas	2.099	2.034 - 2.183
	Colorado	2.732	2.638 - 2.856
	Connecticut	0.174	0.168 - 0.179
	Delaware	0.183	0.175 - 0.188
	Florida1	0.906	0.876 - 0.943
	Georgia	1.085	1.065 - 1.111
	Illinois	2.659	2.593 - 2.710
	Indiana	2.250	2.190 - 2.296
	Iowa	3.497	3.363 - 3.598
	Kansas	2.733	2.671 - 2.814
	Kentucky	1.481	1.424 - 1.525
	Louisiana	1.631	1.630 - 1.631
	Maine	0.676	0.648 - 0.697
	Maryland	0.848	0.813 - 0.874
	Massachusetts	0.361	0.347 - 0.372
	Michigan	4.406	4.263 - 4.516
	Minnesota	13.307	13.082 - 13.602
	Mississippi	1.091	1.080 - 1.100
	Missouri	2.470	2.383 - 2.537
	Montana	1.110	1.050 - 1.188
	Nebraska	2.244	2.218 - 2.264
	New Hampshire	0.232	0.222 - 0.238
	New Jersey	0.364	0.351 - 0.374
	New Mexico	2.508	2.425 - 2.616
	New York	1.742	1.674 - 1.793
	North Carolina	1.168	1.136 - 1.191
	North Dakota	0.808	0.806 - 0.810
	Ohio	2.039	1.954 - 2.104
	Oklahoma	1.745	1.697 - 1.782
	Pennsylvania	1.400	1.343 - 1.444
	Rhode Island	0.135	0.130 - 0.139
	South Carolina	1.373	1.345 - 1.411
	South Dakota	3.411	3.350 - 3.492
	Tennessee	0.797	0.781 - 0.809
	Texas	5.755	5.636 - 5.910
	Vermont	0.228	0.219 - 0.235
	Virginia	1.110	1.069 - 1.142
	West Virginia	0.605	0.578 - 0.626
	Wisconsin	4.074	3.896 - 4.211
	Wyoming	2.561	2.488 - 2.655
Mexico	Guanajuato	0.689	0.661 - 0.711
	Tamaulipas	1.043	0.982 - 1.122
	Michoacán	0.256	0.246 - 0.246
	Querétaro	0.144	0.138 - 0.148
	Nuevo León	0.121	0.116 - 0.124
	Coahuila	0.012	0.011 - 0.012

1Because a large proportion of Monarchs in southern Florida breed and overwinter locally, we excluded this region.


-------------------------------------------------------------------------------------------------------------
Appendix 3: Figures
----------------------------------------------------------------------------------------------------

Figure S1 Distribution of reported Monarch sightings in eButterfly dataset. There are 5,092 unique sightings available in this dataset and their spatial range is limited to Canada and the US.


Figure S2 Pre-migration distribution of adult Monarch butterflies across North America based on scenario 1 (a-c) and scenario 3 (d-f). The figures on the left (a,d) show the relative abundances based on the mean number of Monarchs predicted per pixel, as a proportion of the total of all mean values across all pixels. The figures in the middle (b,e) and right (c,f) show the relative abundances based on the minimum (b,e) and maximum (c,f) number of Monarchs predicted per pixel from the lower and upper limits of the CI for each pixel, as a proportion of the total of all minimum (b,e) and maximum (c,f) values across all pixels.
